# Some Serious Modes of Onset in Pulmonary Tuberculosis

**Published:** 1900-12-08

**Authors:** 


### SOME SERIOUS MODES OF ONSET IN PULMONARY TUBERCULOSIS.

In the first of the Harveian Lectures delivered before the Harveian Society Dr. Robert Maguire dealt with the subject of prognosis in pulmonary tuberculosis, and in so doing he described three modes of onset of this disease which are of especially grave omen. In the first place he insisted upon the gravity of any case of phthisis which begins at more than one point, saying that we may at once decide that the more numerous the initial lesions the more unfavourable must be the prognosis. The bad prognosis of multiple initial lesions has its basis, he says, not merely in the extent of the damage done to the lung tissue, but even more in the indication so given of the intensity of the tuberculous poison and of the vulnerability of the tissues.

In some such cases the patient after a short period of ill-health, accompanied usually by fever of some kind, comes under the notice of the doctor because his friends think that there is something wrong rather than that he thinks so himself. He only complains of a vague languor and of flying aches and pains which might be, and indeed often are, ascribed to rheumatism or neuralgia. Even on the most careful physical examination one may find nothing but a pleuritic rub here and there. There may seem to be no consolidation and no respiratory distress, yet these symptoms are most significant of a powerful tuberculous attack with weak resistance, and they also show that the attack is widely spread. "I know of no beginning," says Dr. Maguire, "which bears a more unfavourable prognosis."

Another most serious form of onset is what has been described by Dr. Mitchell Bruce as "emphysematous phthisis." It is acute both in its onset and its course. The patient complains simply of cough, choking in character, great difficulty of breathing, and on physical examination nothing can be found but the ordinary signs of emphysema. The sputum is bronchitic, but tubercle bacilli may be discovered in it. The temperature may be normal, but sometimes it is raised irregularly, and meanwhile the patient wastes and becomes weaker, until at last there comes an outbreak of physical signs, patches of pneumonia, and finally breakdown of both lungs, and death. This is one of the most serious modes of onset in phthisis.

There is, however, yet another bad form of onset to be considered, one in which we may imagine that the resistance of the patient to the poison is so slight that physical signs are not developed in proportion to the virulence of the infection. It has to be remembered that the extent of the physical signs in any individual case indicates not the intensity of the tuberculous poison by which the system is being invaded, but rather the reaction of the tissues to such an invasion—the "chemiotaxis" as it is called by modern pathologists. The symptoms, on the other hand, are produced by the products of the invasion—to a certain extent, it is true, by the over-action of the body which is involved in the chemiotaxis—but to a greater extent by the poisonous action of the tubercle bacillus and its helping germs. Thus it may be stated as a useful guide, although one of the nature of rule of thumb, that "if the symptoms are out of all proportion greater than the physical signs, think badly of the future of the patient." In such a case the action of the bacillus and its poison is in advance of the resisting power of the lung tissue. One meets cases in which for even a considerable period the symptoms are very marked—so severe indeed as to keep the patient confined to bed for months. The temperature is raised regularly, irregularly, or intermittently, emaciation is continuous, appetite disappears, and weakness is extreme. There may be no cough, or such as may be present is so inconsiderable as to be almost overlooked, while no matter how carefully the physician may examine the chest he cannot find more than the most unimportant physical signs. There may be a little œdematous crackling at the borders of the lungs, an occasional bronchitic sibilus, or even a few streaks of blood, which may be attributed to a congested throat—and these are all. Such a case may be attributed to persistent influenza, not infrequently to subacute rhematism, and sometimes to sheer neurotism. But beware of offering a favourable prognosis in such a case, for this apparent quiescence of lung disease will probably give place after a time to a furious outbreak of lung destruction leading to an early death. This condition may be called "latent pulmonary tuberculosis." The tuberculosis is active enough, but its pulmonary characteristic is latent, and thus it differs from the pleuritic and emphysematous forms already mentioned. Here then we have three modes of onset which are very distinct from the ordinary forms of phthisis in which physical signs and general symptoms go on side by side, showing at the same time a progressive implication of lung tissue, and a coincident deterioration of the system.

